# Supplementary material for: Qualitative exploration of factors associated with COVID-19 vaccination among pregnant women in Kenya
Source: PLOS Glob Public Health. 2026 Feb 18;6(2):e0005927. doi: 10.1371/journal.pgph.0005927 (PMC12915903; doi:10.1371/journal.pgph.0005927)
Supplement: S1 File — (DOCX) [file pgph.0005927.s001.docx]

#### **S1 File. SAGE** **vaccine hesitancy matrix of determinants**

| **Factors** | **Specific determinants** |
| --- | --- |
| **Contextual influences** | a. Communication and media environment b. Influential leaders, immunization program gatekeepers, and anti-or pro-vaccination lobbies  c. Historical influences  d. Religion/culture/gender/socio-economic  e. Politics/policies  f. Geographic barriers  g. Perception of the pharmaceutical industry |
| **Individual and group influences** | a. Personal, family, and /or community members’ experience with vaccination, including pain b. Beliefs, attitudes about health and prevention  c. Knowledge awareness  d. Health system and providers' trust and personal experience  e. Risk/benefit (perceived, heuristic)  f. Immunization as a social norm vs. not needed/harmful |
| **Vaccine/Vaccination specific issues** | a. Risk/Benefit (epidemiological and scientific evidence) b. Introduction of a new vaccine or new formulation or a new recommendation for an existing vaccine  c. Mode of administration  d. Design of vaccination program/mode of delivery (e.g., routine program or mass vaccination campaign)  e. Reliability and/or source of supply of vaccine and/or vaccination equipment  f. Vaccination schedule  g. Costs  h. The strength of the recommendation, and/or knowledge base, and/or attitude of healthcare professionals |

**Source:** SAGE Working Group World Health Organization. Report of the SAGE Working Group on Vaccine Hesitancy, November 2014.
