# Supplementary material for: Qualitative exploration of factors associated with COVID-19 vaccination among pregnant women in Kenya
Source: PLOS Glob Public Health. 2026 Feb 18;6(2):e0005927. doi: 10.1371/journal.pgph.0005927 (PMC12915903; doi:10.1371/journal.pgph.0005927)
Supplement: S2 File — (DOCX) [file pgph.0005927.s002.docx]

**S2 File. Semi-structured interview guides**

**Tool 1. Semi-structured interview guide for pregnant women**

**General questions**

I’d like to hear a little bit about you. Where are you from? Where do you live now?

*Probes*:

- 1. *Do you live near the hospital?*
  2. *Tell me more about your household (e.g. who do you live with?).*

2) Could you tell me your age?

*Probes (if needed):*

1. *Are you less than 30 years old? Older than 50 years*?

Could you tell me about your pregnancy?

*Probes:*

1. *Is this your first pregnancy?*
   - 1. *[If not first pregnancy] How many other children do you have? How old are your children?*
   1. *What trimester are you currently in? [or, for how long have you been pregnant?]*

4) Do you view COVID-19 as an illness that you may come into contact with during your lifetime or not? Why?

*Probes:*

*What have you heard about COVID?*

- - 1. *What have you heard about COVID among pregnant women?*
  1. *Have you or someone you know (e.g., close relative, family member, friend) ever been ill with COVID-19?*
     1. *[if yes] What was your/their experience with COVID-19?*
  2. *Do you know what you can do to protect yourself from COVID? To protect your pregnancy from COVID?*
  3. *Do you think that COVID is a problem in your community? Why or why not?*
  4. *Do you think your community/government has done all it can to protect pregnant women against COVID? Why or why not?*

**Vaccination-related questions**

This next set of questions is about vaccines.

5) I’d like to know more about your experience with vaccines.

*Probes:*

- 1. *Have you ever been vaccinated?*

*[If yes]*

- - 1. *Do you remember which vaccines you received?*
    2. *Do you recall when you received these vaccines?*
    3. *Do you recall why you received these vaccines?*
    4. *What was that experience like?*

*[If no]*

- - 1. *Have you ever been offered a vaccine and decided not to be vaccinated? Why?*
  1. *Have you been vaccinated during your pregnancy?*
     1. *[If not first pregnancy] What about during previous pregnancies? What made you decide to be vaccinated (or not vaccinated)?*
     2. *[If yes] Tell me about your experience getting vaccinated during pregnancy.*

What have you heard about the COVID vaccine?

*Probes:*

- 1. *What have you heard about giving the COVID vaccine to pregnant women?*
  2. *What have doctors, community health workers, or community health volunteers told you about the COVID vaccine for pregnant women?*
  3. *Do you get information about the COVID vaccine from media or social media? If so, tell me about which sources you rely on.*

7) What have you heard about any recommendations related to the COVID vaccine for pregnant women?

*Probes:*

1. *[If participant has heard about it] Where did you learn about this recommendation? The government? Your doctor? Social media? Somewhere else?*
2. *[If participant has not heard of a recommendation or is unsure] What do you think the recommendation is for pregnant women? Why do you think this? How do you feel about this recommendation?*
3. *Is the COVID vaccine currently available for pregnant women in your community?*
   - 1. *[If yes] Do you know which COVID vaccines are available for pregnant women?*
4. *In your opinion, should pregnant women in your community get the COVID vaccine? Why or why not?*

8) Let’s talk about how your family and community feel about COVID vaccines.

*Probes:*

1. *Tell me a little bit about what you think your community feels about COVID-19 vaccines.*
2. *Do you know if your family members have been vaccinated or plan to be vaccinated? Members of your community?*
3. *What percentage of pregnant women in your community do you think are vaccinated (or planning to be vaccinated) for COVID-19?*

Now I’d like to talk about your own experience with COVID vaccines.

9) Have you received the COVID vaccine or do you plan to get that COVID vaccine for yourself? Why or why not?

*Probes:*

*[If yes]*

- 1. *What was the most important factor in your decision to get the COVID vaccine?*
  2. *What information do [did] you need to make an informed decision?*
     1. *Who would [did] you talk to, if anyone?*
     2. *What questions would [did] you ask, if any?*
  3. *When did [or would] you get the COVID vaccine? Why?*
     1. *Before your current pregnancy? During a specific trimester of your pregnancy?*
  4. *Do you know which COVID vaccine you received? Did you have a choice about which vaccine you received?*

*[if yes] Would [did] you prefer a specific COVID vaccine? [(Moderna (Spikevax), Pfizer/BioNTech (Comirnaty), Gamaleya (Sputnik V), Johnson & Johnson/Janssen, AstraZeneca/Oxford (Vaxzevria), Sinopharm (Covilo)]*

- - 1. *Why or why not?*
  1. *What are your concerns, if any, about pregnant women getting the COVID vaccine?*

*[If no – not vaccinated or do not plan to be vaccinated]*

- 1. *What was the most important factor in your decision to not get the COVID vaccine?*
  2. *What information did you need to make an informed decision?*
     1. *Who did you talk to, if anyone?*
     2. *What questions did you ask, if any?*
  3. *Is there anything that would make you more likely to get vaccinated? Please explain.*
  4. *What are your concerns, if any, about pregnant women getting the COVID vaccine?*

*10) [If responded yes to question 9]* Could you tell me a bit more about your experience with getting the vaccine? How difficult or easy was it?

*Probes:*

*a. How did your spouse feel about you getting the vaccine? Your mother-in-law? Other family or friends?*

- - 1. *How much did their opinion affect your decision to get the COVID vaccine?*
  1. *Are there any challenges you faced when trying to get the vaccine? If so, what were they?*
     1. *Access to healthcare, distance and time to clinic, cost of transport, supply/availability, cost/willingness to pay, the need to have a male escort to a clinic, childcare?*
  2. *Where in your community could [did] you get the COVID vaccine? Where could you get more information about the COVID vaccine?*

*11) [If responded no to question 9]* If you wanted to get the COVID vaccine for yourself, how confident do you feel in your ability to get the vaccine?

*Probes:*

- - - - 1. *How would your spouse feel about you getting the vaccine? Your mother-in-law? Other family or friends?*

*How much would their opinion affect your decision to get the COVID vaccine?*

- 1. *Are there any challenges you would face when trying to get the vaccine? If so, what are they?*
     1. *Access to healthcare, distance and time to clinic, cost of transport, supply/availability, cost/willingness to pay, the need to have a male escort to a clinic, childcare?*
  2. *Where in your community could you get the COVID vaccine? Where could you get more information about the COVID vaccine?*

*12) [for all participants]* Is there anything else you would like to tell me about your experience with COVID-19 and the vaccine?

Thank you so much for your time.

**Tool 2. Semi-structured interview guide for women in the post-pregnancy period**

**Study Title:** Exploring knowledge, attitudes, and practices related to COVID-19 vaccine decision-making among pregnant women in Kenya

**General questions**

1. I’d like to hear a little bit about you. Where are you from? Where do you live now?

*Probes*:

- 1. *Do you live near the hospital?*
  2. *Tell me more about your household (e.g. who do you live with?).*

1. Could you tell me your age?

*Probes (if needed):*

1. *Are you less than 30 years old? Older than 50 years*?
2. Could you tell me about your pregnancy?

*Probes:*

- 1. *Tell us about your pregnancy.*

*[If the pregnancy ended in a live birth, proceed with probes b & c then move to question 4. If the pregnancy did not end in a live birth, skip to Question 4]*

- 1. *Is this your first child?*
  2. *Can you tell me the ways in which you are feeding your child (e.g., lactating, using milk substitute)?*

1. Do you view COVID-19 as an illness that you may come into contact with during your lifetime or not? Why?

*Probes:*

- 1. *What have you heard about COVID?*
     1. *What have you heard about COVID among women who have recently been pregnant?*
  2. *Have you ever been ill with COVID-19 or has someone you know (e.g., close relative, family member, friend) ever been ill with this disease?*
     1. *[if yes] What was your/their experience with COVID-19?*
  3. *Do you know what you can do to protect yourself from COVID? [Ask only if pregnancy ended in a live birth] To protect your baby from COVID?*

*Note to interviewer: Empathise with the respondent and consider their emotional state before asking the following questions, taking note that respondent may associate COVID-19 infection with the pregnancy loss. Be careful to skip questions or terminate this line of interviewing if respondent appears to be uncomfortable.*

*_ You informed me that your pregnancy did not end as expected. (Use word of the respondent – miscarriage or stillbirth etc). Are you okay if I ask you some questions following that outcome? (proceed only if the respondent is happy to continue).*

*Do you know what you can do to protect your child from COVID? Do you think the death of your child might have had something to do with COVID? If yes, how so?*

- 1. *Do you think that COVID is a problem in your community? Why or why not?*
  2. *Do you think your community/government has done all it can to protect recently pregnant women against COVID? Why or why not?*

**Vaccination-related questions**

This next set of questions is about vaccines.

1. I’d like to know more about your experience with vaccines.

*Probes:*

- 1. *Have you ever been vaccinated?*

*[If yes]*

- - 1. *Do you remember which vaccines you received?*
    2. *Do you recall when you received these vaccines?*
    3. *Do you recall why you received these vaccines?*
    4. *What was that experience like?*

*[If no]*

- - 1. *Have you ever been offered a vaccine and decided not to be vaccinated? Why?*
  1. *Have you been vaccinated during your pregnancy?*
     1. *[If not first pregnancy] What about during previous pregnancies? What made you decide to be vaccinated (or not vaccinated)?*
     2. *[If yes] Tell me about your experience getting vaccinated during pregnancy.*

1. What have you heard about the COVID vaccine?

*Probes:*

- 1. *What have you heard about giving the COVID vaccine to women who are pregnant? What about for women who have recently been pregnant and are breastfeeding?*
  2. *What have doctors, community health workers, or community health volunteers told you about the COVID vaccine for women who are pregnant? What have they told you about the COVID vaccine for women who have recently given birth and are breastfeeding?*
  3. *Do you get information about the COVID vaccine from media or social media? If so, tell me about which sources you rely on. [Probe with the various types; WhatsApp, Facebook, Instagram, Twitter, Radio, Television]*

1. What have you heard about any recommendations related to the COVID vaccine for women who were recently pregnant?

*Probes:*

1. *[If participant has heard about it] Where did you learn about this recommendation? The government? Your doctor? Social media? Somewhere else?*
2. *[If participant has not heard of a recommendation or is unsure] What do you think the recommendation is for recently pregnant women? Why do you think this? How do you feel about this recommendation?*
3. *Is the COVID vaccine available for women who were recently pregnant in your community? For women who are breastfeeding? What about for women who are not breastfeeding?*
   - 1. *[If yes] Do you know which vaccines are available for women who were recently pregnant? What about for women who are breastfeeding?*
4. *In your opinion, should pregnant women in your community get the COVID vaccine? Why or why not?*
5. Let’s talk about how your family and community feel about COVID vaccines.

*Probes:*

- 1. *Tell me a little bit about what you think your community feels about COVID-19 vaccines.*
  2. *Do you know if your family members have been vaccinated or plan to be vaccinated? Members of your community?*
  3. *What percentage of pregnant women in your community do you think are vaccinated (or planning to be vaccinated) for COVID-19?*

Now I’d like to talk about your own experience with COVID vaccines.

1. Have you received the COVID vaccine for yourself? Why or why not?

*Probes*

*[if they indicated they’ve been vaccinated against COVID at any point]:*

- 1. *What was the most important factor in your decision to get the COVID vaccine?*
  2. *What information did you need to make an informed decision?*
     1. *Who did you talk to, if anyone?*
     2. *What questions did you ask, if any?*
  3. *When did you get your most recent dose of the COVID vaccine? Why?*
     1. *Before pregnancy? A specific trimester during pregnancy? After pregnancy?*
  4. *Do you recall which vaccine you received? Were you given a choice of vaccines?*
     1. *[if yes] Did you have a preference for which vaccine you received? [(Moderna (Spikevax), Pfizer/BioNTech (Comirnaty), Gamaleya (Sputnik V), Johnson & Johnson/Janssen, AstraZeneca/Oxford (Vaxzevria), Sinopharm (Covilo)] Why or why not?*
  5. *To your knowledge, have you received all the doses you are supposed to receive? Why or why not? Follow up: Do you plan to receive all the doses you are supposed to receive? Why or why not*
  6. *What were/are your concerns, if any, about recently pregnant or breastfeeding women getting the COVID vaccine? [Consider avoiding this probe if woman has experienced an unwanted loss of pregnancy]*

*[if they indicated they have NOT been vaccinated against COVID at any point]:*

- 1. *What was the most important factor in your decision to not get the COVID vaccine?*
  2. *What information did you need to make an informed decision?*
     1. *Who did you talk to, if anyone?*
     2. *What questions did you ask, if any?*
  3. *Do you plan to get it now that you are no longer pregnant?*
     1. *[if yes] If given a choice, would you have a preference for which COVID vaccine you receive? [Moderna (Spikevax), Pfizer/BioNTech (Comirnaty), Gamaleya (Sputnik V), Johnson & Johnson/Janssen, AstraZeneca/Oxford (Vaxzevria), Sinopharm (Covilo)] Why or why not?*
     2. *[If yes] What influenced your plans to get the vaccine?*
     3. *[if no] Is there anything that would make you more likely to get vaccinated? Please explain.*
  4. *What are your concerns, if any, about recently pregnant or breastfeeding women getting the COVID vaccine?*

1. *[if they indicated they’ve been vaccinated against COVID at any point]* Could you tell me a bit more about your experience with getting the vaccine? How difficult or easy was it?

*Probes:*

- 1. *How did your spouse feel about you getting the vaccine? Your mother-in-law? Other family or friends?*
     1. *How much did their opinion affect your decision to get the COVID vaccine?*
  2. *Are there any challenges you faced when trying to get the vaccine? If so, what are they?*
     1. *Access to healthcare, distance and time to clinic, cost of transport, supply/availability, cost/willingness to pay, the need to have a male escort to a clinic, childcare?*
  3. *Where in your community did you get the COVID vaccine? Where could you go to get more information about the COVID vaccine, if anywhere?*
  4. *If you needed an additional dose of COVID vaccine, how confident do you feel in your ability to get the vaccine now that you are no longer pregnant?*

1. *[if they indicated they have NOT been vaccinated against COVID at any point]* If you wanted to get the COVID vaccine for yourself, how confident do you feel in your ability to get the vaccine?
   1. *How would your spouse feel about you getting the vaccine? Your mother-in-law? Other family or friends?*
      1. *How much would their opinion affect your decision to get the COVID vaccine?*
   2. *Are there any challenges you would face when trying to get the vaccine? If so, what are they?*
      1. *Access to healthcare, distance and time to clinic, cost of transport, supply/availability, cost/willingness to pay, the need to have a male escort to a clinic, childcare?*
   3. *Where in your community could you get the COVID vaccine? Where could you go to get more information about the COVID vaccine, if anywhere?*
2. *[for all participants]* Is there anything else you would like to tell me about your experience with COVID-19 and the vaccine?

Thank you so much for your time.
