# Supplementary material for: Qualitative exploration of factors associated with COVID-19 vaccination among pregnant women in Kenya
Source: PLOS Glob Public Health. 2026 Feb 18;6(2):e0005927. doi: 10.1371/journal.pgph.0005927 (PMC12915903; doi:10.1371/journal.pgph.0005927)
Supplement: Attachment — Submitted filename: Review paper for COVID19.docx [file pgph.0005927.s005.docx]

**Title: Qualitative exploration of factors associated with COVID-19 vaccination among pregnant women in Kenya**

**General comment:** This is a valuable, well-motivated study with strong fieldwork and clear relevance for maternal immunization programs..

1. **Title –** It is specific and reflecting the context of the manuscript
2. **Abstract**
   a. Abstract/results text mentioned ‘five vaccination-specific issues’ however table 1 in the result section adds the sixth ‘vaccination equipment’
3. **Introduction**
   a. Comprehensive and policy-relevant
   b. Avoid repetition
   c. Add a brief of conceptual map of how SAGE domains map to maternity care pathways
4. **Methodology**
   a. Sampling and saturation – targeting 50 IDIs and balanced trimesters but don’t report about how thematic saturation was assessed?
   b. Inclusion criteria bias of “knowledge of the COVID-19 vaccine” as an eligibility criterion likely skews away from the least informed, who are often the most hesitant. Acknowledge as a limitation and justify.
   c. Translation – quality controls – back-translation, double-checking for idioms – and how Kiswahili nuance were preserved
5. **Results**
   a. Inconsistent counts – postpartum parity 36% primiparous and 56% multiparous which is 92% - what about the remaining 8% missing/unknown?
   b. Participant characteristics table – helping readers assess transferability
   c. Under-developed sub-themes – mode of administration or vaccine equipment
6. **Discussion**
   a. Temporal context matters - the data (Oct 2023–Mar 2024) are post-policy shift (Kenya recommending vaccination in pregnancy) – which should be explicit mentioned in the discussion and tighten interpretation of ‘online self-registration barriers’
   b. Actionable segmentation converting segmented recommendations
      i. Pregnant women
      ii. Postpartum
      iii. Low-resource settings
   c. Several paragraphs restate results – include more synthesize
      i. Link misinformation to trusted intermediary solutions
      ii. Link transport to service-design fixes
      iii. Link booster hesitancy to risk communication
7. **Study Limitations and strengthens**
   a. Selection bias
   b. Social desirability
   c. Recall bias
   d. Language + translation limitation
8. **Conclusion and recommendations**
   a. Concise and appropriate
9. **References**
   a. Avoid over-reliance on media sources for policy milestones – cite MoH and WHO technical docs
   b. Duplicate citations 48 & 49
10. **Others**
    a. Terminology – use postpartum consistently (avoid post pregnancy) + healthcare workers

**Decision:** Minor revision
