# Supplementary material for: Qualitative exploration of factors associated with COVID-19 vaccination among pregnant women in Kenya
Source: PLOS Glob Public Health. 2026 Feb 18;6(2):e0005927. doi: 10.1371/journal.pgph.0005927 (PMC12915903; doi:10.1371/journal.pgph.0005927)
Supplement: Attachment — Submitted filename: Response to reviewers.docx [file pgph.0005927.s007.docx]

Dear Editor

Re: PGPH-D-25-02543

**Qualitative exploration of factors associated with COVID-19 vaccination among pregnant women in Kenya**

Thank you for reviewing our manuscript and for the invitation to submit a revised version that addresses the points raised during the review process.
I have attached a marked-up copy of the manuscript that highlights changes made to the original version (Revised Manuscript with Track Changes) and an unmarked version without tracked changes (Manuscript).

**Editor’s comments**

1. Please provide a detailed online Financial Disclosure statement. This is published with the article. It must therefore be completed in full sentences and contain the exact wording you wish to be published.

a) Please clarify all sources of financial support for your study. List the grants, grant numbers, and organizations that funded your study, including funding received from your institution. Please note that suppliers of material support, including research materials, should be recognized in the Acknowledgements section rather than in the Financial Disclosure. 
b) State the initials, alongside each funding source, of each author to receive each grant.

For example: “This work was supported by the National Institutes of Health (####### to AM; ###### to CJ) and the National Science Foundation (###### to AM).”
c) State what role the funders took in the study. If the funders had no role in your study, please state: “The funders had no role in study design, data collection and analysis, decision to publish, or preparation of the manuscript.”

For more information, please go to our submission guidelines:
<https://journals.plos.org/globalpublichealth/s/submission-guidelines#loc-financial-disclosure-statement>

**Response:** This work was supported by the Bill & Melinda Gates Foundation grant INV-016877 to R. Limaye/ R.Karron. The funders had no role in study design, data collection and analysis, decision to publish, or preparation of the manuscript.

2. Please ensure that the funders and grant numbers match between the Financial Disclosure field and the Funding Information tab in your submission form. Note that the funders must be provided in the same order in both places as well.

**Response:** This has been addressed in the submission form.

3. In this instance it seems there may be acceptable restrictions in place that prevent the public sharing of your minimal data. However, in line with our goal of ensuring long-term data availability to all interested researchers, PLOS’ Data Policy states that authors cannot be the sole named individuals responsible for ensuring data access (<http://journals.plos.org/globalpublichealth/s/data-availability#loc-acceptable-data-sharing-methods>).

Data requests to a non-author institutional point of contact, such as a data access or ethics committee, helps guarantee long term stability and availability of data. Providing interested researchers with a durable point of contact ensures data will be accessible even if an author changes email addresses, institutions, or becomes unavailable to answer requests.

Before we proceed with your manuscript, please also provide non-author contact information (phone/email/hyperlink) for a data access committee, ethics committee, or other institutional body to which data requests may be sent. If no institutional body is available to respond to requests for your minimal data, please consider if there any institutional representatives who did not collaborate in the study, and are not listed as authors on the manuscript, who would be able to hold the data and respond to external requests for data access? If so, please provide their contact information (i.e., email address). Please also provide details on how you will ensure persistent or long-term data storage and availability.

**Response:**

Our revised data availability statement reads as follows: All relevant data are within the paper, and data will be made available to interested researchers on request. All requests can be made to the AKU Kenya Research Office, Aga Khan Institutional Scientific and Ethics Committee, Email: AKUKenya.ResearchOffice@aku.edu

4. If the reviewer comments include a recommendation to cite specific previously published works, please review and evaluate these publications to determine whether they are relevant and should be cited. There is no requirement to cite these works unless the editor has indicated otherwise.

**Response:** Thank you. We have reviewed and added two new relevant references.

5. Please review your reference list to ensure that it is complete and correct. If you have cited papers that have been retracted, please include the rationale for doing so in the manuscript text, or remove these references and replace them with relevant current references. Any changes to the reference list should be mentioned in the rebuttal letter that accompanies your revised manuscript. If you need to cite a retracted article, indicate the article’s retracted status in the References list and also include a citation and full reference for the retraction notice.

**Response:** Thank you. We have reviewed our reference list accordingly

6. Please revise accordingly. Please consider the INTERCIVID experience it is a multinational consortium producing major evidence on this end.

**Response:** It is not clear what you are referring to as ‘the INTERCIVID experience.’ Kindly clarify.

**REVIEWER 1**

**General comment:** This is a valuable, well-motivated study with strong fieldwork and clear relevance for maternal immunization programs.

**Title –** It is specific and reflecting the context of the manuscript

**Response:** Thank you.

**Abstract**

Abstract/results text mentioned ‘five vaccination-specific issues’ however table 1 in the result section adds the sixth ‘vaccination equipment’

**Response:** Thank you very much for that edit. We have deleted ‘vaccination equipment’ from Table 1. Page 6.

**Introduction**

Comprehensive and policy-relevant

Avoid repetition

Add a brief of conceptual map of how SAGE domains map to maternity care pathways

**Response:** Thank you, we have avoided repetition. In this paper, we do not address maternity care pathways. The SAGE mapping that we refer to in this paper is the WHO Strategic Advisory Group of Experts on Immunization (SAGE) and its definition of vaccine hesitancy. We have added a brief reference to it in the introduction. Page 3.

**Methodology**

Sampling and saturation – targeting 50 IDIs and balanced trimesters but don’t report about how thematic saturation was assessed?

**Response:** Thank you for this observation. During the study design, the research team intentionally considered the sample size needed to collect adequate data to inform the research questions. We were guided by past research, see e.g. 1. Sample sizes for saturation in qualitative research: A systematic review of empirical tests, <https://www.sciencedirect.com/science/article/pii/S0277953621008558>; 2. Characterising and justifying sample size sufficiency in interview-based studies: systematic analysis of qualitative health research over a 15-year period <https://bmcmedresmethodol.biomedcentral.com/articles/10.1186/s12874-018-0594-7>; 3. Sample Sizes for 10 Types of Qualitative Data Analysis: An Integrative Review, Empirical Guidance, and Next Steps

<https://journals.sagepub.com/doi/10.1177/16094069241296206>;

4. Saturation: An Overworked and Misunderstood Concept?

<https://journals.sagepub.com/doi/10.1177/10778004231183948?icid=int.sj-full-text.similar-articles.2>; 5. What Influences Saturation? Estimating Sample Sizes in Focus Group Research

<https://journals.sagepub.com/doi/10.1177/1049732318821692?icid=int.sj-full-text.similar-articles.3>.

We thus worked with an intentional sample size of 50 IDIs, and as we coded data the 50^th^ transcript, there were no new insights emerging from our data.

Inclusion criteria bias of “knowledge of the COVID-19 vaccine” as an eligibility criterion likely skews away from the least informed, who are often the most hesitant. Acknowledge as a limitation and justify.

**Response:** Thank you. We have acknowledged this in the study limitations (Page 24). Kindly note that the recruitment of those who had knowledge of the vaccine was intentional, and this is why our tool consisted of vaccination-related questions. See Page 5.

Translation – quality controls – back-translation, double-checking for idioms – and how Kiswahili nuance were preserved

**Response:** Translation from Swahili to English was carried out by competent assistants to ensure preservation of Kiswahili idioms and nuances – it was not a literal translation from English. All fifty audio files were transcribed and translated by research assistants who were fluent in English and Swahili. Page 5

**Results**

a. Inconsistent counts – postpartum parity 36% primiparous and 56% multiparous which is 92% - what about the remaining 8% missing/unknown?

**Response:** Thank you for this observation. We have added a new Table 1 capturing the socio-demographics of the study participants. Page 6. Of the 25 postpartum participants, 36% were primiparous and 64% had more than one child.

b. Participant characteristics table – helping readers assess transferability

**Response:** We have added a new Table 1 capturing the socio-demographics of the study participants. Page 6.

c. Under-developed sub-themes – mode of administration or vaccine equipment

**Response:** Thank you for this observation. The sub-theme noted in our data on mode of administration, and we reported it on Page 17. We have deleted ‘vaccination equipment’ from Table 2 (the old Table 1). Page 7 – apologies for the confusion caused.

**Discussion**

a. Temporal context matters - the data (Oct 2023–Mar 2024) are post-policy shift (Kenya recommending vaccination in pregnancy) – which should be explicit mentioned in the discussion and tighten interpretation of ‘online self-registration barriers’

**Response:** Thank you. We have captured the temporal context of our work accordingly. Page 20.

b. Actionable segmentation converting segmented recommendations

Pregnant women

Postpartum

Low-resource settings

**Response:** Added in the conclusion section, Page 23.

c. Several paragraphs restate results – include more synthesize

Link misinformation to trusted intermediary solutions

Link transport to service-design fixes

Link booster hesitancy to risk communication

**Response:** We have included more synthesis. Page 18-20.

**Study Limitations and strengthens**

a. Selection bias

b. Social desirability

c. Recall bias

d. Language + translation limitation

**Response:** Thank you. We have revised the study limitations and strengths. Page 22.

**Conclusion and recommendations**

Concise and appropriate

**Response:** Thank you.

**References**

Avoid over-reliance on media sources for policy milestones – cite MoH and WHO technical docs

Duplicate citations 48 & 49

**Response:** Duplicate citations have been removed, and we have avoided over-reliance on media sources.

**Others**

Terminology – use postpartum consistently (avoid post pregnancy) + healthcare workers

**Response:** We have checked for consistent use. Thank you.

**Decision:** Minor revision

**Response:** Thank you for your review.

**REVIEWER 2**

This research article entitled “Qualitative exploration of factors associated with COVID-19 vaccination among pregnant women in Kenya” provides valuable information pregnant and postpartum women in relation with COVID-19 vaccination. The study design and methodology are robust and address an important public health issue. The manuscript is well-written. However, here are some comments to improve their quality:

1. The manuscript needs a further literature review to include current evidence on this topic.
For example, there are other COVID-19 vaccine studies conducted with pregnant and postpartum women in Kenya, that have not been referenced, such as:
Marwa MM, et al. COVID-19 vaccine hesitancy among pregnant and postpartum Kenyan women. Int J Gynaecol Obstet. 2023 Jul;162(1):147-153. doi: 10.1002/ijgo.14773. Epub 2023 Apr 10. PMID: 37036449; PMCID: PMC10330087.
Ayieko S, Jaoko W, Opiyo RO, Orang'o EO, Messiah SE, Baker K, Markham C. Knowledge, Attitudes, and Subjective Norms Associated with COVID-19 Vaccination among Pregnant Women in Kenya: An Online Cross-Sectional Pilot Study Using WhatsApp. Int J Environ Res Public Health. 2024 Jan 16;21(1):98. doi: 10.3390/ijerph21010098. PMID: 38248561; PMCID: PMC10815556.

**Response:** Thank you. We have reviewed the evidence and included the two citations above.

2. Methods: Why didn’t you include interviews with the partners? I understand that is key to have women’s perspectives, but children care is responsibility of both parents (in case of pregnant women who are within a relationship, that one could guess that is most of cases). Partners’ views, as well as family views, are key for decision-making in vaccine acceptability for pregnant women. This is well reflected in the limitations section.

**Response:** Thank you for this observation. We have captured the limitation accordingly. Page 22.

3. Results: Please, review and strengthen the first paragraph of the Results section.

**Response:** We have reviewed, thank you. Page 6.

4. Results: I would encourage authors to include a “Table 1” with sociodemographic characteristics of participants in the study. It would be valuable to see not only the basic sociodemographic factors, but the number of women who accepted COVID-19 and other vaccines during pregnancy.

**Response:** Thank you for this proposal. We have included a new Table 1 capturing the characteristics of study participants. Page 6.

5. Results: Within a quote you included the “HCP” acronym, however it was not been defined, nor used throughout the manuscript. I would encourage you to use the acronym HCW or HCP, if not, to please, delete that and include the full definition of the term.

**Response:** Thank you for this observation. We have provided it in full. Page 18.

6. Discussion: It would be worth to discuss a bit more the role of the fake news, and miscommunication. For example, some participants declared some fears that lead to non vaccination, such as vaccination affecting erectile function, fertility, population control etc.

**Response:** We have included more discussion of these findings. Pages 18, 20.

7. Results: I would have expected some results discussing the role of spirituality, religion, or religious leaders in vaccine acceptance or hesitancy, as it was briefly mentioned in the discussion section that it was one of the factors affecting other studies. Did you explore this within the data?
**Response:** Thank you for this idea – indeed, spirituality and religion influence health behaviors. We did not discuss these dimensions in depth as our participants did not raise them.
