# Supplementary material for: Qualitative exploration of factors associated with COVID-19 vaccination among pregnant women in Kenya
Source: PLOS Glob Public Health. 2026 Feb 18;6(2):e0005927. doi: 10.1371/journal.pgph.0005927 (PMC12915903; doi:10.1371/journal.pgph.0005927)
Supplement: Attachment — Submitted filename: Response_to_reviewers_auresp_2.docx [file pgph.0005927.s008.docx]

Dear Editor

Re: PGPH-D-25-02543R1

**Qualitative exploration of factors associated with COVID-19 vaccination among pregnant women in Kenya**

Thank you for reviewing our manuscript again and for the invitation to submit a revised version that addresses the points raised during the second review process.

We have attached a marked-up copy of the manuscript that highlights changes made (Revised Manuscript with Track Changes) and an unmarked version without tracked changes (Manuscript).

**Editor’s comments**

Journal Requirements:

If the reviewer comments include a recommendation to cite specific previously published works, please review and evaluate these publications to determine whether they are relevant and should be cited. There is no requirement to cite these works unless the editor has indicated otherwise.

Please review your reference list to ensure that it is complete and correct.

**Response:** Thank you. We have reviewed our reference list accordingly, and added useful citations.

**Additional Editor Comments:**

The revision was successful. However before publication I believe some work should be done to reinforce the concept that vaccine is beneficial for mothers, fetuses and neonates. Particularly the authors may enrich the background with knowledge from major studies showing that neonates from vaccinated mothers have lower risks. Neonates of vaccinated mothers had a decreased risk for preterm birth and adverse neonatal outcomes. (1)

More so the authors should support the effort in preventing vaccine hesitancy as stated in recent opinions and major work (2) Please refer them to major guidelines of other coutries and international consortia if the trust in local authorities is low. Please add this concepts in order to reinforce vaccine uptake locally, this is critical to improve the outcome.

1. Barros FC, et al. ; INTERCOVID-2022 International Consortium. Maternal vaccination against COVID-19 and neonatal outcomes during Omicron: INTERCOVID-2022 study. Am J Obstet Gynecol. 2024 Oct;231(4):460.e1-460.e17. doi: 10.1016/j.ajog.2024.02.008. Epub 2024 Feb 16. PMID: 38367758.

2. Cavoretto PI, Farina A. Time to enhance COVID-19 vaccination in women of reproductive age. Lancet Reg Health Eur. 2024 Sep 10;45:101069. doi: 10.1016/j.lanepe.2024.101069. PMID: 39308776; PMCID: PMC11415629.

**Response:** Thank you. We have reviewed the evidence and included the two citations above, and one additional reference.

**Reviewers' comments:**

**Reviewer #1:**

The authors have addressed the comments appropriately.

**Response:** Thank you very much for your review.

**Reviewer #2:**

Thank you for addressing the comments from the first round of peer review. My only remaining comment is that you need to update the COREQ checklist. Please make sure to indicate the page numbers in the manuscript where each piece of information is provided, rather than adding explanations or repeating content. For example, in the cell where you listed “NVIVO,” you should instead specify the page number in the manuscript where the use of this software is described. The same applies to items such as the derivation of themes and other methodological details, etc. Please update the checklist accordingly.

**Response:** Thank you very much for your review. We have updated the COREQ Checklist to fully align with our current manuscript.

**Other comments**

Figure Resubmissions:

While revising your submission, we strongly recommend that you use PLOS’s NAAS tool (https://ngplosjournals.pagemajik.ai/artanalysis) to test your figure files.

**Response:** We do not have any figures in our submission. Thank you.

Sincerely,

VIOLET NAANYU (on behalf of all authors)
